# RNA-seq reveals transcriptome changes in goats following myostatin gene knockout

**DOI:** 10.1371/journal.pone.0187966

**Published:** 2017-12-11

**Authors:** Lamei Wang, Bei Cai, Shiwei Zhou, Haijing Zhu, Lei Qu, Xiaolong Wang, Yulin Chen

**Affiliations:** 1 College of Animal Science and Technology, Northwest A&F University, Yangling, China; 2 Shaanxi Provincial Engineering and Technology Research Center of Cashmere Goats, Yulin, China; 3 Life Science Research Center, Yulin University, Yulin, China; China Agricultural University, CHINA

## Abstract

Myostatin (MSTN) is a powerful negative regulator of skeletal muscle mass in mammalian species that is primarily expressed in skeletal muscles, and mutations of its encoding gene can result in the double-muscling trait. In this study, the CRISPR/Cas9 technique was used to edit *MSTN* in Shaanbei Cashmere goats and generate knockout animals. RNA sequencing was used to determine and compare the transcriptome profiles of the muscles from three wild-type (WT) goats, three fibroblast growth factor 5 (*FGF5*) knockout goats (FGF5^+/-^ group) and three goats with disrupted expression of both the *FGF5* and *MSTN* genes (FM^+/-^ group). The sequence reads were obtained using the Illumina HiSeq 2000 system and mapped to the *Capra hircus* reference genome using TopHat (v2.0.9). In total, 68.93, 62.04 and 66.26 million clean sequencing reads were obtained from the WT, FM^+/-^ and FGF5^+/-^ groups, respectively. There were 201 differentially expressed genes (DEGs) between the WT and FGF5^+/-^ groups, with 86 down- and 115 up-regulated genes in the FGF5^+/-^ group. Between the WT and FM^+/-^ groups, 121 DEGs were identified, including 81 down- and 40 up-regulated genes in the FM^+/-^ group. A total of 198 DEGs were detected between the FGF5^+/-^ group and FM^+/-^ group, with 128 down- and 70 up-regulated genes in the FM^+/-^ group. At the transcriptome level, we found substantial changes in genes involved in fatty acid metabolism and the biosynthesis of unsaturated fatty acids, such as stearoyl-CoA dehydrogenase, 3-hydroxyacyl-CoA dehydratase 2, ELOVL fatty acid elongase 6 and fatty acid synthase, suggesting that the expression levels of these genes may be directly regulated by *MSTN* and that these genes are likely downstream targets of *MSTN* with potential roles in lipid metabolism in goats. Moreover, five randomly selected DEGs were further validated with qRT-PCR, and the results were consistent with the transcriptome analysis. The present study provides insight into the unique transcriptome profile of the *MSTN* knockout goat, which is a valuable resource for studying goat genomics.

## Introduction

Myostatin (MSTN) is a secreted growth factor and a member of the TGF-β superfamily that functions as a critical autocrine/paracrine inhibitor and negatively regulates skeletal muscle growth and development through the regulation of anabolic and catabolic pathways in skeletal muscles [1]. Myostatin is expressed almost exclusively in skeletal muscle [2]. Mutations in the coding region of *MSTN* result in a double-muscling phenotype in many species, including cattle [3, 4], mice [5–7] and humans [8]. Myostatin both regulates lean tissue mass and affects body fat content in mice [9]. In *MSTN* knockout mice (*MSTN*^*-/-*^), the absence of *MSTN* results in increased skeletal muscle mass, reduced fat tissue, increased insulin sensitivity, enhanced fatty acid oxidation, and an elevated resistance to obesity [6, 10], whereas overexpression of myostatin induces muscle atrophy [11]. Consequently, a number of strategies to block the effects of myostatin have been developed and tested in various models of neuromuscular disorders, muscle-wasting conditions or metabolic disturbances [12–14]. Mice with Lewis Lung carcinoma treated with ActRIIB-Fc, a soluble myostatin receptor that binds myostatin, showed increased body weight and muscle weight as well as significantly increased grip strength [15]. In *mdx* mice, a model for Duchenne muscular dystrophy, an antibody-mediated myostatin blockade was found to ameliorate the pathophysiology and muscle weakness associated with this condition [16]. However, these strategies have less efficiency in blocking the effects of myostatin.

Natural myostatin gene mutations occur in cattle breeds such as the Belgian Blue, which exhibits obviously increased muscle mass [17]. Muscular hypertrophy, or the double-muscle phenotype, is a heritable condition in cattle [3]. The Shaanbei Cashmere goat is a local breed in China that produces both fiber and meat products. The meat is renowned for its lower fat content compared to that of beef and lamb [18], and thus, it is recommended as a healthy food for consumers. To increase meat production in goats, efforts aimed at the development of strategies to significantly increase muscle growth by manipulating *MSTN* gene expression have been intensively undertaken [19].

High-throughput mRNA sequencing (RNA-seq) offers the ability to discover new genes and transcripts and to measure both transcript abundance and expression in a single experiment [20, 21]. Based on RNA-seq technology, we measured the integrated global gene expression and signaling pathway activities in goats after *MSTN* gene knockout. Microarray technology enables a broad overview of the impact of treatments on the expression of all known genes, and this technology has been already been used to examine the effects of constitutive *MSTN* deficiency in mice and cattle [22–25]. However, high-throughput mRNA sequencing (RNA-Seq) offers the ability to discover new genes and transcripts and to measure transcript expression levels in a single assay [26–28]. Many studies have shown that RNA-Seq is more accurate over a greater dynamic range of gene expression than expression microarrays [29, 30]. Recently, genome-wide gene expression profiling has gained ground due to the development and application of large-scale sequencing techniques to obtain gene sequences and develop molecular markers, especially in less researched species [31–34].

Wang et al. recently reported the successful application of the CRISPR/Cas9 system to manipulate the goat genome [35]. They also demonstrated the utility of this approach by disrupting *MSTN*, which resulted in enhanced body weight and larger muscle fibers in goats with Cas9-mediated genetic modifications. They further characterized the effects of these genome modifications by hematoxylin and eosin (H&E) staining, quantitative PCR, Western blotting, and immunofluorescence staining (Animal Genetics, under review). However, a transcriptomic analysis of the changes in either gene expression or lipid metabolism in *MSTN* knockout goats has not been reported.

*MSTN* was initially characterized as a potent inhibitor of skeletal muscle growth and development [36]. However, its role in the regulation of glucose and lipid metabolism has gained significant attention [9, 37–40]. The goals of this study were to identify important candidate genes related to muscle, glucose and lipid metabolism and to further determine the transcriptomic changes using RNA-Seq. These results will provide valuable information about key genes in this species and improve our understanding of the molecular mechanisms regulating muscle, glucose and lipid metabolism in goats.

## Materials and methods

### Ethics statement

All animal experiments and procedures were carried out in strict accordance with the recommendations of the Guide for the Care and Use of Laboratory Animals by the National Institutes of Health. The protocol was approved by the Committee on the Ethics of Animal Experiments of Northwest A&F University (Approval ID: 2014ZX08008-002).

### Animal samples

The animals used in the present study were previously generated by Wang et al. [35]. The genotypes of three *MSTN*- and *FGF5*-disrupted goats and three *FGF5*-disrupted goats are summarized in S1 Table. All animals were raised in the same way under natural light with free access to water and food at the Shaanbei Cashmere Goat Farm at Yulin University. Longissimus dorsi muscles were obtained from nine one-year-old Shaanbei Cashmere goats: three wild-type (WT) goats (each was determined to possess an unedited genome), three goats with only the *fibroblast growth factor 5* (*FGF5*) gene knocked out (FGF5^+/-^ group) and three goats with disruptions in both the *FGF5* and *MSTN* genes (FM^+/-^ group). The transgenic goats were generated using the CRISPR/Cas9 system. Fresh longissimus dorsi muscle samples were harvested immediately from goats after surgery under local anesthesia using 3 mL of procaine (1%) injected into the skin/muscle tissues. All efforts were made to minimize animal suffering and to reduce the number of animals used. Obtained fresh longissimus dorsi muscle samples were washed with sterile normal saline three times, frozen in RNAlater (Takara, Dalian, China) using liquid nitrogen, and sent to Novogene Bioinformatics Technology Co. Ltd. (Beijing, China).

### RNA extraction and preparation

Genome-wide transcriptome libraries were constructed from the three sets of muscle samples from the WT, FGF5^+/-^ and FM^+/-^ goats. Longissimus dorsi muscle tissue blocks containing approximately 80–100 mg of tissue were dissolved in TRIzol reagent (Invitrogen, USA) for total RNA extraction. RNA degradation and contamination were monitored by electrophoresis in 1% agarose gels. The quality and quantity of RNA were checked using a NanoPhotometer® spectrophotometer (IMPLEN, CA, USA) and a Qubit® RNA Assay Kit in a Qubit® 2.0 Fluorometer (Life Technologies, CA, USA). RNA integrity was assessed using an RNA Nano 6000 Assay Kit and a Bioanalyzer 2100 system (Agilent Technologies, CA, USA).

### mRNA-Seq library preparation and sequencing

Illumina® (NEB, USA) mRNA-Seq libraries were prepared with 3 μg of total RNA using the NEBNext® Ultra™ RNA Library Prep Kit according to the manufacturer’s instructions, and index codes were added to attribute the sequences to each sample. Briefly, RNA was purified from total RNA using Poly-T oligo-attached magnetic beads. Fragmentation was carried out using divalent cations under elevated temperature conditions in NEBNext First Strand Synthesis Reaction Buffer (5x). First-strand cDNA was synthesized using random hexamer primers and M-MLV Reverse Transcriptase (RNase H minus). Second-strand cDNA synthesis was subsequently performed using DNA Polymerase I and RNase H. Remaining overhangs were converted into blunt ends via exonuclease/polymerase activities. After adenylation of the 3’ ends of cDNA fragments, a NEBNext Adaptor with a hairpin loop structure was ligated to prepare the cDNA for hybridization. To preferentially select cDNA fragments of 150~200 bp in length, the library fragments were purified with the AMPure XP system (Beckman Coulter, Beverly, USA). Then, 3 μL of USER Enzyme (NEB, USA) was used with size-selected, adaptor-ligated cDNA at 37°C for 15 min followed by 5 min at 95°C before PCR. Then, PCR was performed with Phusion High-Fidelity DNA polymerase, Universal PCR primers and Index (X) Primer. Finally, the PCR products were purified (AMPure XP system), and the library quality was assessed using the Agilent Bioanalyzer 2100 system.

Clustering of the index-coded samples was performed on a cBot Cluster Generation System using the TruSeq PE Cluster Kit v3-cBot-HS (Illumina) according to the manufacturer’s instructions. After cluster generation, the library preparations were sequenced on an Illumina HiSeq 2000 platform, and 100-bp paired-end reads were generated.

### Quality control and read mapping to the reference genome

Raw data (raw reads) in the FASTQ format were first processed using in-house Perl scripts and the Q20, Q30 and GC contents of the clean data were calculated.

For mapping, an index of the reference genome was built using Bowtie v2.0.6, and paired-end clean reads were aligned to the reference genome using TopHat v2.0.9.

### Gene expression

The counts of the read numbers mapped to each gene were processed by HTSeq v0.6.1, and the FPKM (expected number of fragments per kilobase sequence per million base pairs sequenced) of each gene was calculated based on the length of the gene and the reads count mapped to that gene [41]. The FPKM considers both the effect of sequencing depth and gene length for the read count and is currently the most commonly used method for estimating gene expression levels [42].

DEGs were identified using the R package “DESeq” (1.10.1) with raw gene counts as the input, and quantile normalization was applied for variable library sizes. DESeq provides statistical routines for determining differential expression in digital gene expression data using a model based on the negative binomial distribution. The resulting *P*-values were adjusted using the Benjamini and Hochberg’s approach for controlling the false discovery rate [43]. Differentially expressed genes (DEGs) were determined by DEGseq with a cutoff threshold of *P*-value < 0.05 [44].

### Differential gene functional annotation

Gene Ontology (GO) and Kyoto Encyclopedia of Genes and Genomes databases (KEGG, http://www.genome.jp/kegg) enrichment was analyzed using differential genes as the foreground and all genes as the background for the identified differential transcripts. GO enrichment analysis of the DEGs was performed using the GOseq R package [45], in which gene length bias was corrected. GO terms with corrected *P-*values < 0.05 were considered significantly enriched by DEGs.

The KEGG is a database resource for understanding high-level functions and utilities of the biological system [46]. KEGG Orthology Based Annotation System (KOBAS, 2.0) software was used to test the statistical enrichment of differentially expression genes in the KEGG pathways.

### qRT-PCR analysis for the validation of RNA-Seq data

Real-time quantitative reverse transcription PCR (qRT-PCR) was used to validate the RNA-Seq data. The total RNA isolated for RNA sequencing was used to perform qRT-PCR. Reverse transcription was performed with 1 μg of total RNA using the PrimeScript RT Reagent Kit (Invitrogen, USA). Real-time quantitative PCR analyses were then performed with a Bio-Rad IQ5 Optical System; individual reactions were prepared with 5 ng of cDNA and SYBR Green PCR master mix (Takara, Dalian, China) in a final volume of 20 μL. All reactions were performed in triplicate. Cycle threshold (Ct) values were normalized to glyceraldehyde 3-phosphate dehydrogenase (GAPDH), and comparative quantification of mRNA was performed using the 2^-ΔΔCt^ method. The primer sequences used in the qPCR assay are provided in S2 Table [47–49].

### Statistical analysis

All data are presented as the means ± standard deviation (SD), and comparisons were performed by analysis of variance (ANOVA) (SAS). A probability of less than 0.05 was considered to be statistically significant.

## Results

### Summary of the raw sequence reads and DEGs

To understand the gene expression profiles and differences between the three samples at transcript resolution, we performed RNA-Seq on three cDNA sequencing libraries constructed using muscle tissues from WT, FGF5^+/-^ and FM^+/-^ goats. We then mapped the filtered clean reads to the reference goat genome CHIR-1.0 (Table 1). In total, we obtained 68.93, 62.04 and 66.26 million reads for the sequencing libraries constructed from the WT, FGF5^+/-^ and FM^+/-^ goat muscle tissues, respectively. We found that in each sample, ~73% of reads could be mapped to the reference genome, which is comparable to the reports for two non-model organisms, swine [50] and bovine [51], in which 61.4–75.0% of reads are mapped to the reference genome. Thus, our mapping percentage suggests good sequence quality.

**Table 1:** Statistics of the Illumina RNA-Seq reads in the normal and transgenic goat muscle libraries that were mapped to the goat reference genome CHIR-1.0.

Summary statistics	Number	Percentage	Number	Percentage	Number	Percentage
	WT		FM^+/-^		FGF5^+/-^	
Total raw reads	70553433		63677463		67796573	
Total mapped	51626789	74.99%	45847205	73.90%	48958298	73.90%
Total clean reads	68933544		62036347		66257588	
Total clean base pairs (Gb)	10.34 Gb		9.30 Gb		9.94 Gb	
Read map to '+'	25346942	36.81%	22469249	36.22%	24050354	36.30%
Read map to '-'	25053573	36.39%	22207247	35.80%	23732089	35.83%
Multiply mapped	1226274	1.79%	1170709	1.89%	1175855	1.77%
Uniquely mapped	50400515	73.00%	44676496	72.01%	47782443	72.13%
Non-splice reads	25925041	37.59%	22021886	35.52%	24005700	36.24%
Splice reads	24475474	35.61%	22654610	36.49%	23776743	35.88%

### Identification of DEGs

**Fig 1:**
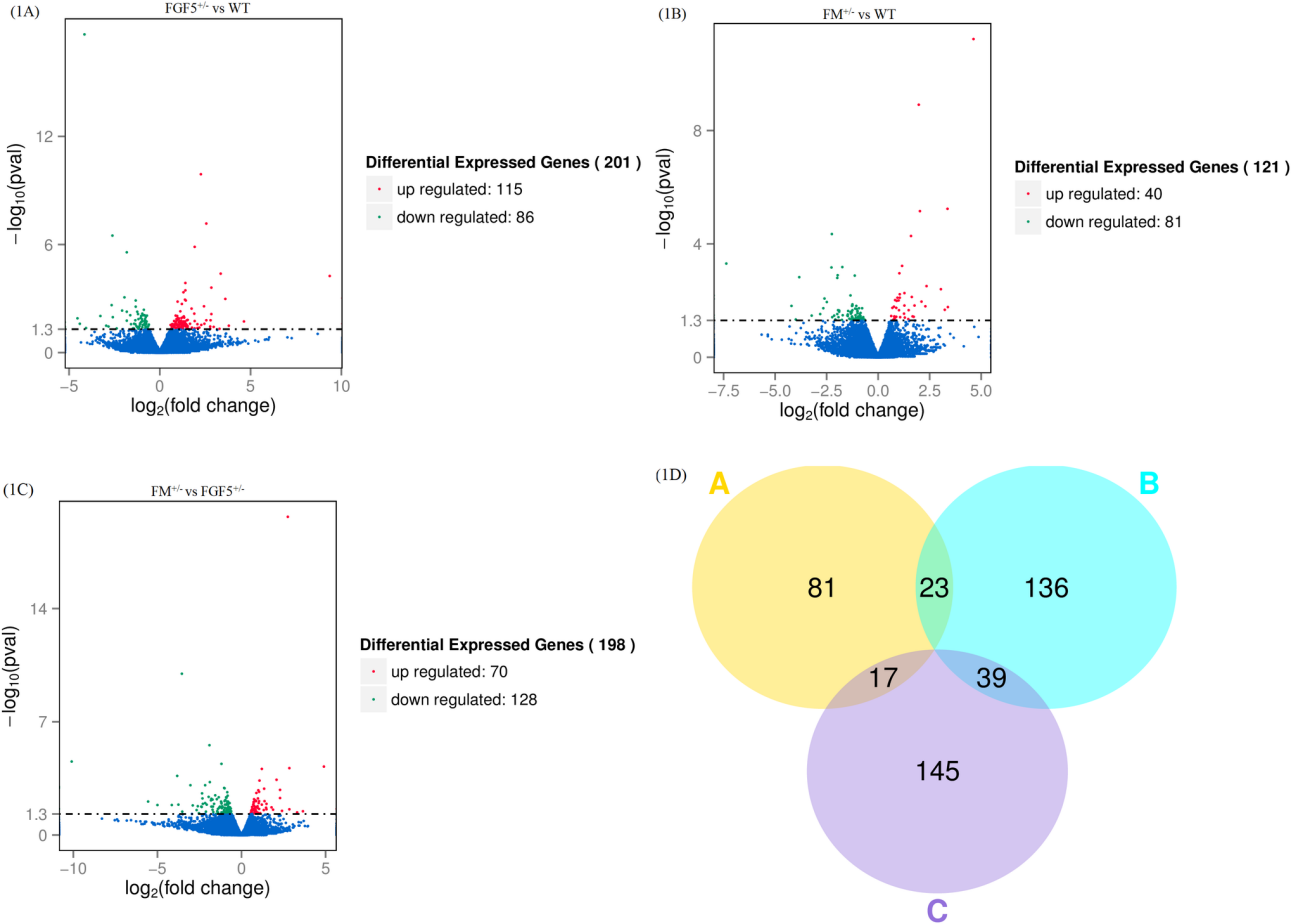
Differentially expressed genes in the RNA-Seq data. Volcano plot of statistically significant differentially expressed genes at P ≤ 0.05 identified from the RNA-Seq libraries of normal and transgenic goat muscle (1A, 1B, 1C). A Venn diagram showing the DEGs identified from comparisons of FGF5^+/-^ vs WT goats, FM^+/-^ vs WT goats and FM^+/-^ vs FGF5^+/-^ goats (1D).

**Fig 2:**
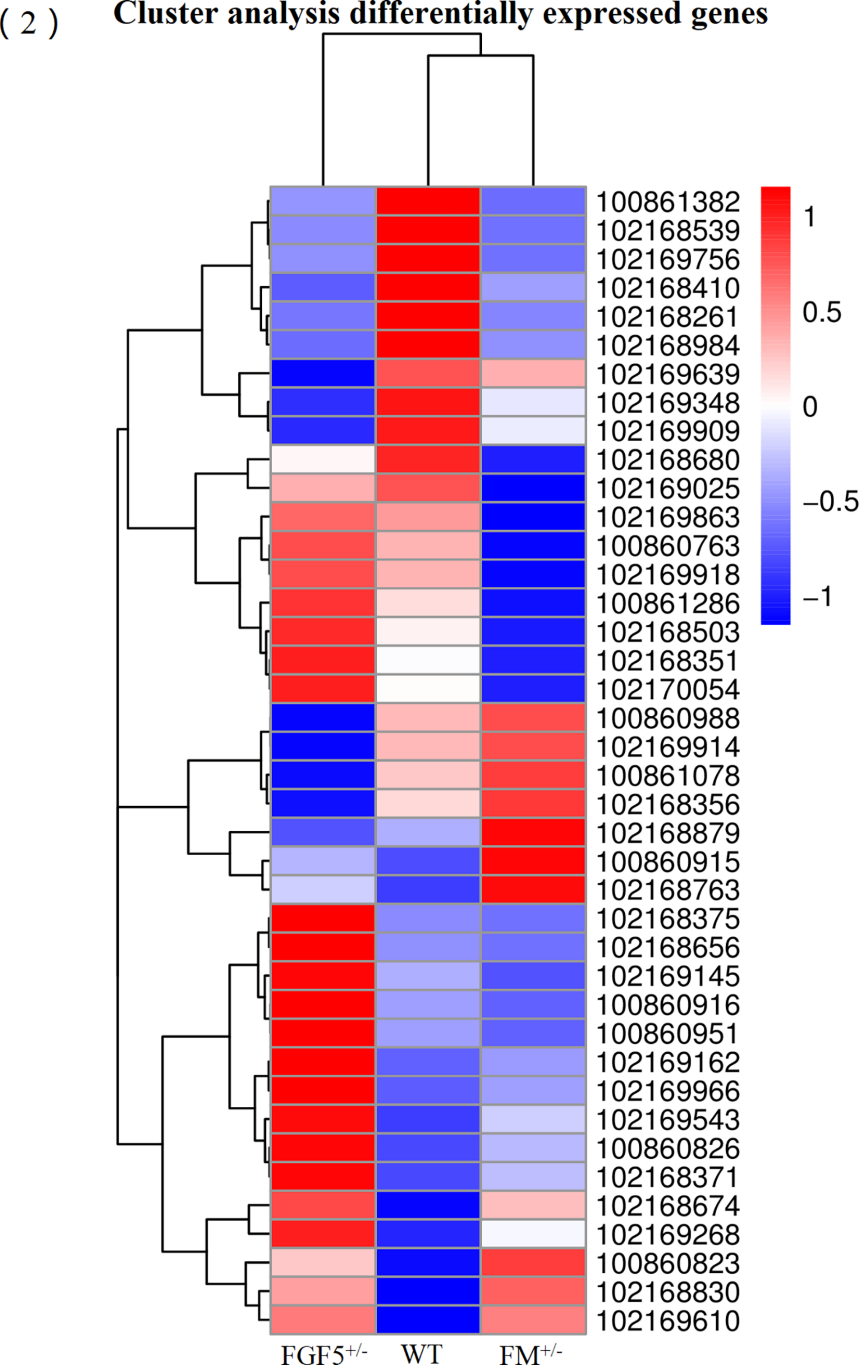
A hierarchically clustered heatmap showing the expression patterns of the 40 most DEGs. The red blocks represent the overexpressed genes, and the blue blocks represent genes with the lowest expression levels. Colored bars indicate the expression levels.

The read count data obtained from the transcriptome were used to analyze differences in gene expression. We obtained 201 DEGs, including 115 up-regulated genes and 86 down-regulated genes, between the FGF5^+/-^ group and the WT group. Moreover, 121 DEGs, including 40 up-regulated genes and 81 down-regulated genes, were identified between the FM^+/-^ group and the WT group. A total of 198 DEGs, including 70 up-regulated genes and 128 down-regulated genes, were identified between the FM^+/-^ group and the FGF5^+/-^ group. We used a false discovery rate (FDR) ≤ 0.001 and an absolute value of the log2 ratio ≥ 1 as the threshold to judge the significance of differences in gene expression. Library size-normalized counts for the three samples were generated, and volcano plots for the DEGs are shown in Fig 1A, 1B and 1C. A Venn diagram showing the number of DEGs among the three pairwise comparisons is shown in Fig 1D. Hierarchical clustering showed the expression profiles of the top 40 DEGs (Fig 2). The full names of the top 20 DEGs in the WT, FM^+/-^ and FGF5^+/-^ goats and their functional characteristics are shown in Table 2 [52–70].

**Table 2:** Biological functions of the 20 most differentially expressed genes in the longissimus dorsi muscle identified by pairwise comparisons of FGF5^+/-^ vs WT, FM^+/-^ vs WT and FM^+/-^ vs FGF5^+/-^.

Gene ID	Symbol	Summary
100861382	KRT27	This gene encodes a member of the type I (acidic) keratin family, which belongs to the superfamily of intermediate filament (IF) proteins that is essential for the proper assembly of type I and type II keratin protein complexes and the formation of keratin intermediate filaments in the inner root sheath (IRS) [52].
102168539	ROR2	The protein encoded by this gene is a receptor protein tyrosine kinase and type I transmembrane protein that belongs to the ROR subfamily of cell surface receptors. The protein may be involved in the early formation of the chondrocytes and may be required for cartilage and growth plate development [53].
102169758	NUDT2	This gene encodes a member of the MutT family of nucleotide pyrophosphatases, a subset of the larger NUDIX hydrolase family. The gene may be a candidate tumor suppressor gene [54].
102168410	GJC3	This gene encodes a gap junction protein. The encoded protein is a connexin that plays a role in the formation of gap junctions, which provide direct connections between adjacent cells [55].
102168261	EPHA7	Activation of the protein encoded by this gene results in activating phosphorylation of components of the ERK signaling pathway, including MAP2K1, MAP2K2, MAPK1 and MAPK3 [56].
102168984	TNMD	The gene tenomodulin (TNMD) is a tendon-specific marker known to be important for tendon maturation, with key implications for the residing tendon stem/progenitor cells and for the regulation of endothelial cell migration in chordae tendineae cordis in the heart and in experimental tumor models [57]. Myostatin has a potential role in the induction of tenogenic differentiation of C2C12 cells [58].
102169639	MIB1	This gene encodes a protein that contains multiple ankyrin repeats and RING finger domains and functions as an E3 ubiquitin ligase. The encoded protein positively regulates Notch signaling by ubiquitinating Notch receptors, thereby facilitating Notch receptor endocytosis. This protein may also promote the ubiquitination and degradation of death-associated protein kinase 1 (DAPK1) [59].
102169348	CDH19	This gene is one of three related type II cadherin genes situated in a cluster on chromosome 18. The encoded protein is a calcium-dependent cell-cell adhesion glycoprotein containing five extracellular cadherin repeats. CDH19 plays important roles in cell adhesion and in the formation of adherens junctions, which bind cells together within tissues [60].
102169909	ARL15	The function of this gene has yet to be established.
102168680	LOC102168680	The function of this gene has yet to be established.
102169025	SLC24A3	Plasma membrane sodium/calcium exchangers play important roles in intracellular calcium homeostasis and electrical conduction. Potassium-dependent sodium/calcium exchangers such as SLC24A3 are believed to exchange 1 intracellular calcium ion and 1 potassium ion for 4 extracellular sodium ions [61].
102169863	EGFL6	This gene encodes a member of the epidermal growth factor (EGF) repeat superfamily. Members of this superfamily are characterized by the presence of EGF-like repeats and are often involved in the regulation of the cell cycle, proliferation, and developmental processes [62].
100860763	SCD	Stearoyl-CoA desaturase (Δ-9-desaturase) is an endoplasmic reticulum enzyme that catalyzes the rate-limiting step in the formation of monounsaturated fatty acids (MUFAs), specifically oleate and palmitoleate, from stearoyl-CoA and palmitoyl-CoA [63].
102169918	HECW2	This gene encodes a member of a family of E3 ubiquitin ligases that plays an important role in the proliferation, migration and differentiation of neural crest cells via the regulation of glial cell line-derived neurotrophic factor (GDNF)/Ret signaling. This gene also plays an important role in angiogenesis by stabilizing endothelial cell-to-cell junctions via the regulation of angiomotin-like 1 stability [64].
100861286	FASN	Fatty acid synthase is a multi-enzyme protein that catalyzes fatty acid synthesis. Its main function is to catalyze the synthesis of palmitate (C16:0), a long-chain saturated fatty acid, from acetyl-CoA and malonyl-CoA in the presence of NADPH [65].
102168503	DOK6	DOK6 is a member of the DOK (see DOK1; MIM 602919) family of intracellular adaptors that plays a role in the RET (MIM 164761) signaling cascade [66].
102168351	TIAM2	This gene encodes a guanine nucleotide exchange factor. A highly similar mouse protein specifically activates ras-related C3 botulinum substrate 1, converting this Rho-like guanosine triphosphatase (GTPase) from an inactive, guanosine diphosphate-bound state to an active, guanosine triphosphate-bound state. The encoded protein may also play a role in neural cell development [67].
102170054	MERTK	This gene encodes a member of the MER/AXL/TYRO3 receptor kinase family. The encoded transmembrane protein contains two fibronectin type-III domains, two Ig-like C2-type (immunoglobulin-like) domains, and one tyrosine kinase domain. Mutations in this gene have been associated with disruption of the retinal pigment epithelium (RPE) phagocytosis pathway and the onset of autosomal recessive retinitis pigmentosa [68].
100860988	TPMT	Thiopurine methyltransferase methylates thiopurine compounds. The methyl donor is S-adenosyl-L-methionine, which is converted to S-adenosyl-L-homocysteine. This enzyme uses S-adenosyl-L-methionine as an S-methyl donor to metabolize thiopurine drugs, producing S-adenosyl-L-homocysteine as a byproduct [69].
102169914	KLHL23	The protein encoded by this gene is a member of the kelch family of proteins, which is characterized by a 44–56 amino acid repeat motif. The kelch motif appears in many different polypeptide contexts and contains multiple potential protein-protein contact sites. Members of this family have diverse activities and are present both throughout the cell and extracellularly [70].

### Functional enrichment of DEGs

The identified DEGs were further analyzed using GO and KEGG enrichment to determine their potential functions and metabolic pathways.

**Fig 3:**
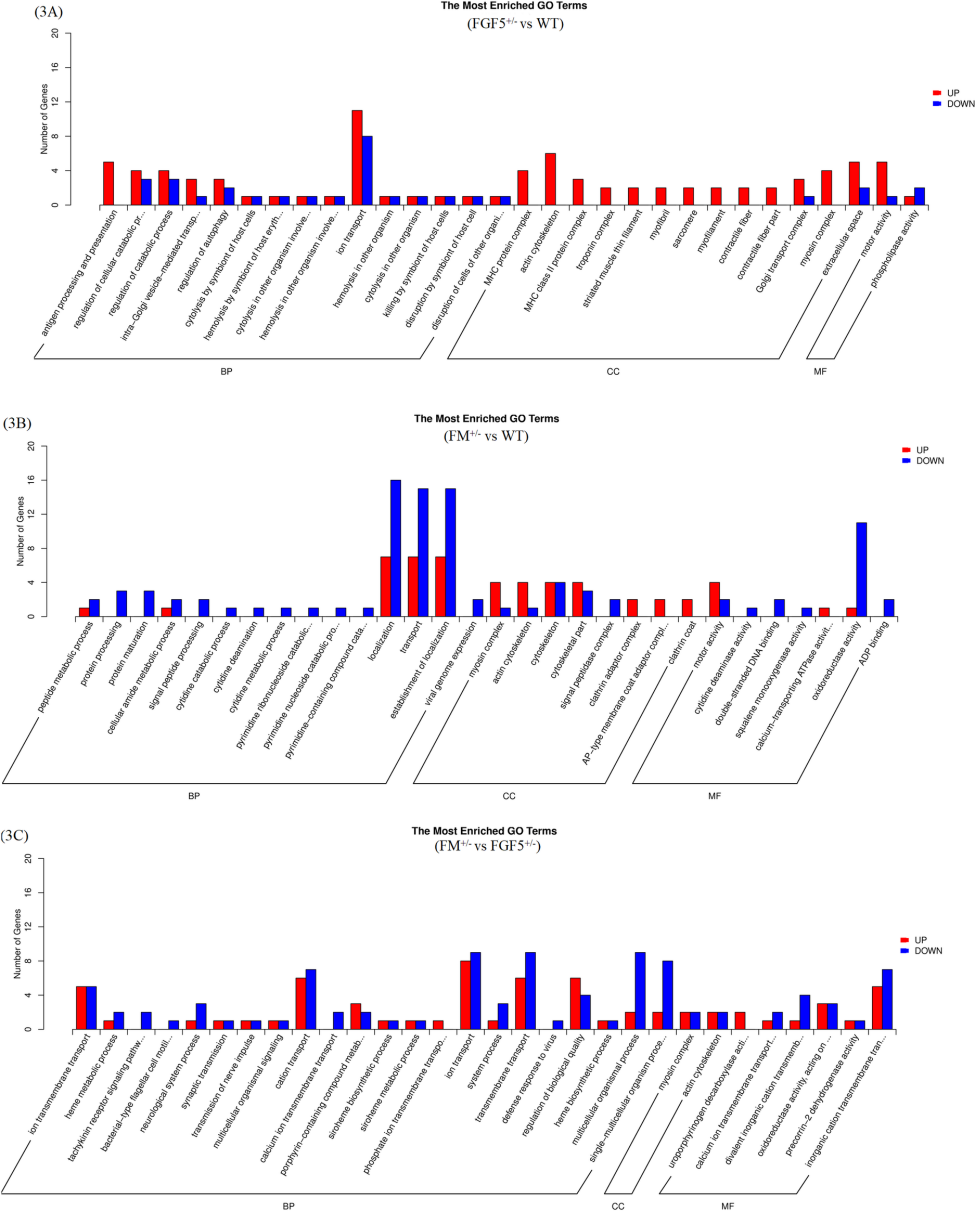
Gene ontology analysis summary. Classification of the annotated amino-acid sequences. Amino-acid sequences were grouped into different functional subcategories: cellular component (CC), molecular function (MF) and biological process (BF). 3A: FGF5^+/-^ vs WT; 3B: FM^+/-^ vs WT; and 3C: FM^+/-^ vs FGF5^+/-^.

GO analysis based on biological process (BP) enrichment was performed for sets of DEGs with significant cluster profiles. Only significant GO categories with *P-*values < 0.05 were chosen for analysis. The results of the GO enrichment analysis of DEGs were classified into three categories: BP, cellular component (CC) and molecular function (MF). Fig 3 shows the GO classifications of the DEGs from the comparisons between FGF5^+/-^ and WT goats, FM^+/-^ and WT goats, and FM^+/-^ and FGF5^+/-^ goats.

The top 20 up- and down-regulated gene-enriched GO terms are shown in Tables 3 and 4. By comparing the gene list of FGF5^+/-^ goats with that of WT goats, two major GO terms relevant to muscle contraction [(actin cytoskeleton and myosin heavy chain (MHC) protein complex)] were identified (Fig 3A). The machinery that powers cell migration is built from the actin cytoskeleton, which is larger than any organelle, and the assembly or disassembly of the actin cytoskeleton can easily change cell morphology [71]. The distribution of enriched GO terms between gene lists of FM^+/-^ and WT goats is shown in Fig 3B. Moreover, the myosin complex and actin cytoskeleton were significantly enriched in the CC. Myosin comprises a superfamily of ATP-dependent motor proteins and is best known for its roles in muscle contraction and its involvement in a wide range of other motility processes in eukaryotes [72]. In terms of BPs, several processes in the fatty acid metabolic system, including peptide metabolism, protein processing and cellular amide metabolism, were found to be enriched in the genes down-regulated in FM^+/-^ goats compared with their expression in WT goats. There were several major enriched GO terms in the comparison of gene lists from FM^+/-^ and FGF5^+/-^ goats (Fig 3C).

**Table 3:** The top 20 GO terms of significantly up-regulated genes.

Treatments	GO ID	Category	GO description	*P*-Value
FGF5^+/-^ vs WT	GO:0015629	cellular component	actin cytoskeleton	4.29E-06
	GO:0042611	cellular component	MHC protein complex	4.45E-05
	GO:0019882	biological process	antigen processing and presentation	7.64E-05
	GO:0016459	cellular component	myosin complex	1.45x10^-4^
	GO:0042613	cellular component	MHC class II protein complex	2.97 x10^-4^
	GO:0003774	molecular function	motor activity	3.38 x10^-4^
	GO:0005861	cellular component	troponin complex	6.65 x10^-4^
	GO:0005865	cellular component	striated muscle thin filament	6.65 x10^-4^
	GO:0030016	cellular component	myofibril	6.65 x10^-4^
	GO:0030017	cellular component	sarcomere	6.65 x10^-4^
	GO:0036379 GO:0043292 GO:0044449	cellular component cellular component cellular component	myofilament contractile fiber contractile fiber part	6.65 x10^-4^ 6.65 x10^-4^ 6.65 x10^-4^
	GO:0005886	cellular component	plasma membrane	1.31x10^-3^
	GO:0044281	biological process	small molecule metabolic process	1.44x10^-3^
	GO:0032182	molecular function	mall conjugating protein bindings	1.72 x10^-3^
	GO:0043130	molecular function	ubiquitin binding	1.72 x10^-3^
	GO:0017119	cellular component	Golgi transport complex	1.96 x10^-3^
	GO:0006206	biological process	pyrimidine nucleobase metabolic process	2.78 x10^-3^
	GO:0019856	biological process	pyrimidine nucleobase biosynthetic process	2.78 x10^-3^
FM^+/-^ vs WT	GO:0016459	cellular component	myosin complex	2.68E-06
	GO:0015629	cellular component	actin cytoskeleton	1.46E-05
	GO:0003774	molecular function	motor activity	6.82E-05
	GO:0030131	cellular component	clathrin adaptor complex	1.14 x10^-3^
	GO:0030119	cellular component	AP-type membrane coat adaptor complex	1.39 x10^-3^
	GO:0030118	cellular component	clathrin coat	1.85 x10^-3^
	GO:0016192	biological process	vesicle-mediated transport	3.12 x10^-3^
	GO:0044430	cellular component	cytoskeletal part	3.62 x10^-3^
	GO:0009755	biological process	hormone-mediated signaling pathway	4.8 x10^-3^
	GO:0032870	biological process	cellular response to hormone stimulus	4.8 x10^-3^
	GO:0071495	biological process	cellular response to endogenous stimulus	4.97 x10^-3^
	GO:0005575	cellular component	cellular component	5.13 x10^-3^
	GO:0005388	molecular function	calcium-transporting ATPase activity	5.18 x10^-3^
	GO:0071310	biological process	cellular response to organic substance	5.29 x10^-3^
	GO:0009725	biological process	response to hormone stimulus	5.29 x10^-3^
	GO:0009719	biological process	response to endogenous stimulus	5.45 x10^-3^
	GO:0005856	cellular component	cytoskeleton	5.95 x10^-3^
	GO:0070887	biological process	cellular response to chemical stimulus	6.49 x10^-3^
	GO:0010033	biological process	response to organic substance	6.67 x10^-3^
	GO:0009399	biological process	nitrogen fixation	6.89 x10^-3^
FM^+/-^ vs FGF5^+/-^	GO:0004853	molecular function	uroporphyrinogen decarboxylase activity	4.70E-05
	GO:0034220	biological process	ion transmembrane transport	1.72 x10^-3^
	GO:0031090	cellular component	organelle membrane	2.2 x10^-3^
	GO:0065008	biological process	regulation of biological quality	2.72 x10^-3^
	GO:0035435	biological process	phosphate ion transmembrane transport	3.82 x10^-3^
	GO:0019725	biological process	cellular homeostasis	4.22 x10^-3^
	GO:0015672	biological process	monovalent inorganic cation transport	5.14 x10^-3^
	GO:0006779	biological process	porphyrin-containing compound biosynthetic process	5.56 x10^-3^
	GO:0042592	biological process	homeostatic process	7.41 x10^-3^
	GO:0006778	biological process	porphyrin-containing compound metabolic process	7.66 x10^-3^
	GO:0006811	biological process	ion transport	8.43 x10^-3^
	GO:0051701	biological process	interaction with host	9.33 x10^-3^
	GO:0004427	molecular function	inorganic diphosphatase activity	9.39 x10^-3^
	GO:0033014	biological process	tetrapyrrole biosynthetic process	0.01
	GO:0005388	molecular function	calcium-transporting ATPase activity	0.01
	GO:0006812	biological process	cation transport	0.01
	GO:0015114	molecular function	phosphate ion transmembrane transporter activity	0.01
	GO:0033013	biological process	tetrapyrrole metabolic process	0.01
	GO:0045454	biological process	cell redox homeostasis	0.01
	GO:0019048	biological process	modulation by virus of host morphology or physiology	0.01

**Table 4:** The top 20 GO terms of significantly down-regulated genes.

Treatments	GO ID	Category	GO description	*P*-Value
FGF5^+/-^ vs WT	GO:0009055	molecular function	electron carrier activity	1.95 x10^-3^
	GO:0019911	molecular function	structural constituent of myelin sheath	3.11 x10^-3^
	GO:0015109	molecular function	chromate transmembrane transporter activity	3.89 x10^-3^
	GO:0015703	biological process	chromate transport	3.89 x10^-3^
	GO:0005198	molecular function	structural molecule activity	7.78 x10^-3^
	GO:0045502	molecular function	dynein binding	9.59 x10^-3^
	GO:0008813	molecular function	chorismate lyase activity	0.01
	GO:0005515	molecular function	protein binding	0.01
	GO:0004620	molecular function	phospholipase activity	0.01
	GO:0042803	molecular function	protein homodimerization activity	0.01
	GO:0019015	cellular component	viral genome	0.01
	GO:0007156	biological process	homophilic cell adhesion	0.01
	GO:0016833	molecular function	oxo-acid-lyase activity	0.02
	GO:0016337	biological process	cell-cell adhesion	0.02
	GO:0003913	molecular function	DNA photolyase activity	0.02
	GO:0005521	molecular function	lamin binding	0.02
	GO:0046983	molecular function	protein dimerization activity	0.02
	GO:0015099	molecular function	nickel cation transmembrane transporter activity	0.03
	GO:0015675	biological process	nickel cation transport	0.03
	GO:0044699	biological process	single-organism process	0.03
FM^+/-^ vs WT	GO:0016485	biological process	protein processing	7.3 x10^-4^
	GO:0051604	biological process	protein maturation	7.3 x10^-4^
	GO:0016491	molecular function	oxidoreductase activity	2.62 x10^-3^
	GO:0005787	cellular component	signal peptidase complex	2.94 x10^-3^
	GO:0006465	biological process	signal peptide processing	2.94 x10^-3^
	GO:0055114	biological process	oxidation-reduction process	4.44 x10^-3^
	GO:0004126	molecular function	cytidine deaminase activity	7.06 x10^-3^
	GO:0006216	biological process	cytidine catabolic process	7.06 x10^-3^
	GO:0009972	biological process	cytidine deamination	7.06 x10^-3^
	GO:0046087	biological process	cytidine metabolic process	7.06 x10^-3^
	GO:0046133	biological process	pyrimidine ribonucleoside catabolic process	7.06 x10^-3^
	GO:0046135	biological process	pyrimidine nucleoside catabolic process	7.06 x10^-3^
	GO:0072529	biological process	pyrimidine-containing compound catabolic process	7.06 x10^-3^
	GO:0003690	molecular function	double-stranded DNA binding	7.11 x10^-3^
	GO:0019080	biological process	viral genome expression	7.86 x10^-3^
	GO:0006518	biological process	peptide metabolic process	9.37 x10^-3^
	GO:004353	molecular function	ADP binding	0.01
	GO:0004506	molecular function	squalene monooxygenase activity	0.01
	GO:0005135	molecular function	interleukin-3 receptor binding	0.02
	GO:0043566	molecular function	structure-specific DNA binding	0.02
FM^+/-^ vs FGF5^+/-^	GO:0007217	biological process	tachykinin receptor signaling pathway	1.27 x10^-3^
	GO:0071973	biological process	bacterial-type flagellar cell motility	4.24 x10^-3^
	GO:0070838	biological process	divalent metal ion transport	4.79 x10^-3^
	GO:0070588	biological process	calcium ion transmembrane transport	5.67 x10^-3^
	GO:0072511	biological process	divalent inorganic cation transport	8.87 x10^-3^
	GO:0051607	biological process	defense response to virus	9.86 x10^-3^
	GO:0032501	biological process	multicellular organismal process	0.01
	GO:0042168	biological process	heme metabolic process	0.01
	GO:0072509	molecular function	divalent inorganic cation transmembrane transporter activity	0.01
	GO:0007601	biological process	visual perception	0.01
	GO:0030553	molecular function	cGMP binding	0.01
	GO:0050953	biological process	sensory perception of light stimulus	0.01
	GO:0003856	molecular function	3-dehydroquinate synthase activity	0.01
	GO:0044707	biological process	single-multicellular organism process	0.01
	GO:0030416	biological process	methylamine metabolic process	0.01
	GO:1901160	biological process	primary amino compound metabolic process	0.01
	GO:0006816	biological process	calcium ion transport	0.01
	GO:0050877	biological process	neurological system process	0.01
	GO:0008113	molecular function	peptide-methionine (S)-S-oxide reductase activity	0.01
	GO:0030551	molecular function	cyclic nucleotide binding	0.01

### Pathway analysis

**Fig 4:**
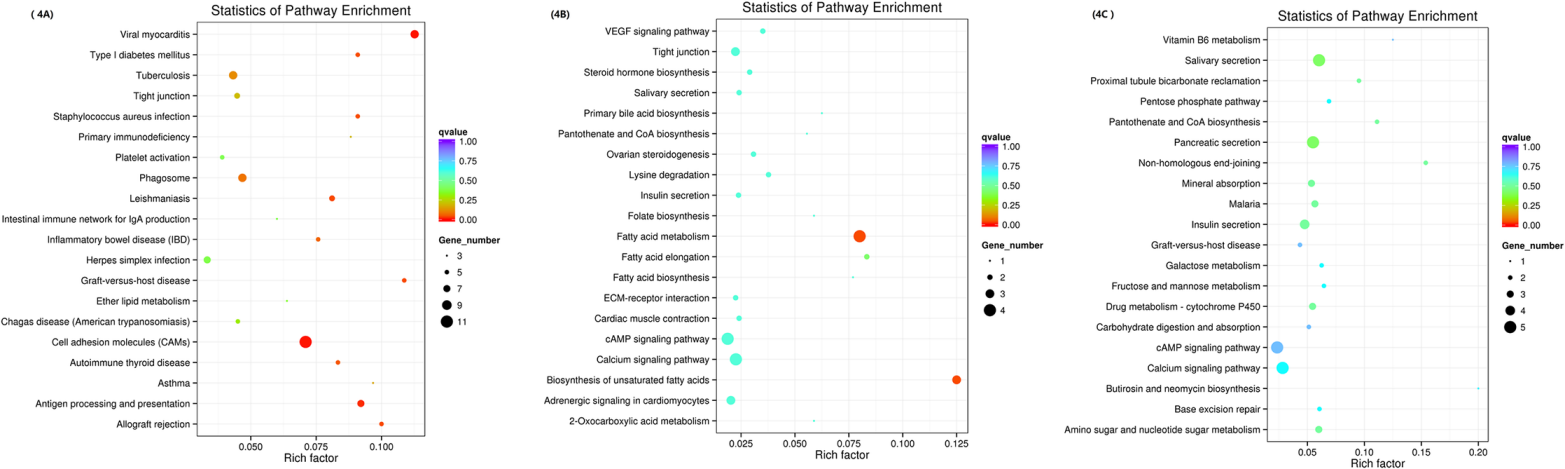
KEGG pathway enrichment analysis. 4A: FM^+/-^ vs WT; 4B: FGF5^+/-^ vs WT. 4C: FM^+/-^ vs FGF5^+/-^.

**Fig 5:**
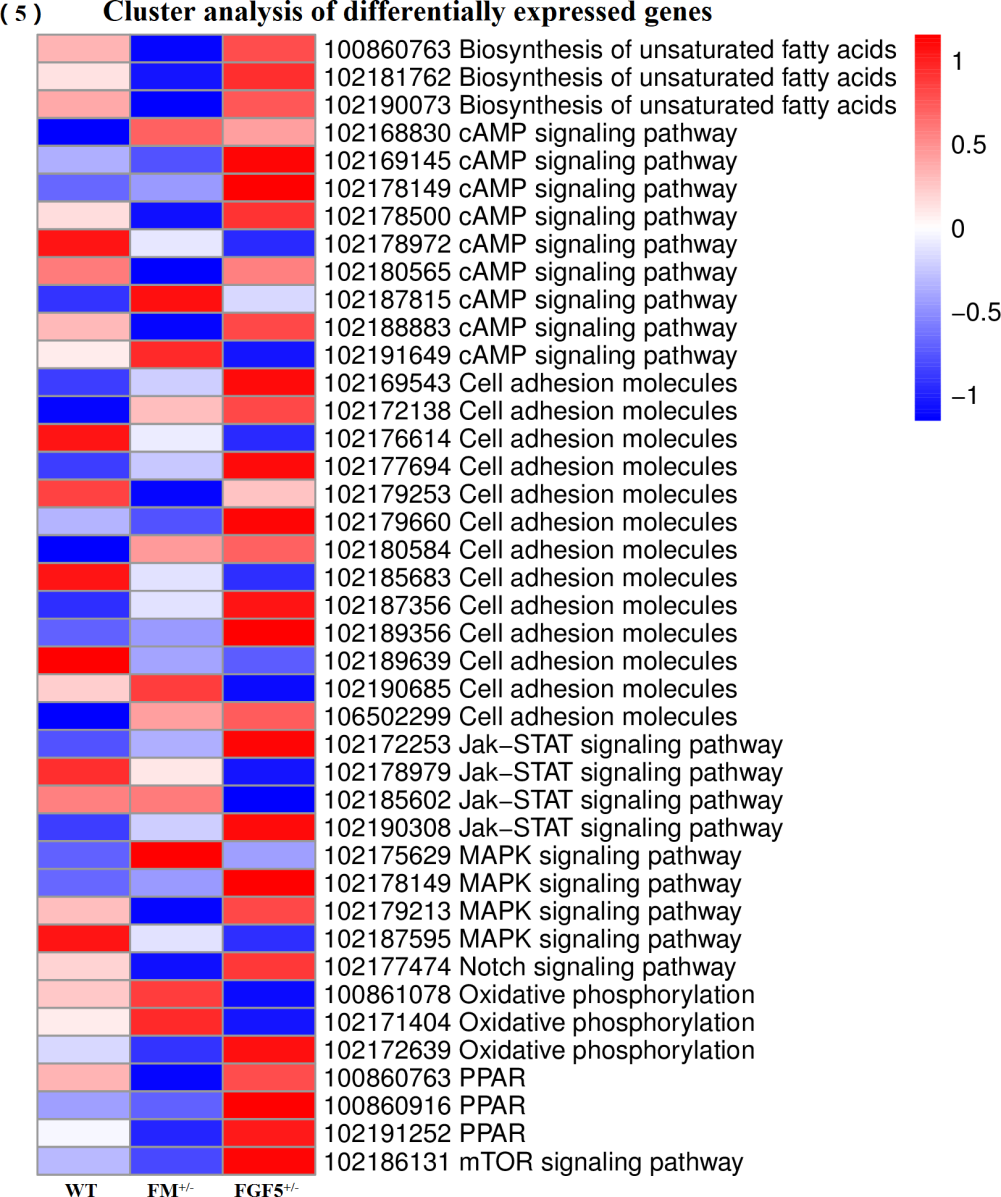
Cluster analysis of DEGs. Heat map of the expression levels of DEGs related to lipid, glucose, protein and oxidative phosphorylation metabolism pathways in the muscles of the goats.

As shown in Fig 4, the top 20 pathways were identified by pairwise comparisons of complete DEGs lists from every group. The results from the KEGG analysis revealed many genes related to fatty acid metabolism, the peroxisome proliferator-activated receptor (PPAR) signaling pathway, the mitogen-activated protein kinase (MAPK) signaling pathway and cell adhesion molecules. Additionally, the KEGG analysis revealed several genes involved in Notch signaling, AMP-activated protein kinase (AMPK) signaling, mTOR-signaling, oxidative phosphorylation, and Jak-STAT signaling. Many genes significantly down-regulated in the FM^+/-^ goats compared to their expression in WT and FGF5^+/-^ goats were associated with fatty acid biosynthesis (*stearoyl-CoA desaturase*, *fatty acid synthase*, *ELOVL fatty acid elongase 6* and *3-hydroxyacyl-CoA dehydratase 2*) and the biosynthesis of unsaturated fatty acids (*stearoyl-CoA desaturase*, *fatty acid synthase* and *3-hydroxyacyl-CoA dehydratase 2*). Genes involved in the PPAR signaling pathway (*stearoyl-CoA desaturase*), MAPK signaling pathway [(*mitogen-activated protein kinase 3*, MAPK3 or ERK1), (ras-related C3 botulism toxin substrate 2, RAC2), cell division cycle 25B (CDC25B), phospholipase A2 group IVE (PLA2G4E)], biosynthesis of unsaturated fatty acids, Notch signaling (*hes family bHLH transcription factor 1* and *HES 1*), AMPK signaling (*calcium binding protein 39-like* and *fructose-bisphosphatase 1*) and mTOR-signaling (*calcium binding protein 39-like*) were also down-regulated. On the other hand, along with an increased percentage of differentially expressed gene sets between FGF5^+/-^ goats and WT goats, the DEGs clustered in the pathways of viral myocarditis, cell adhesion molecules (CAMs), antigen processing and presentation, graft-versus-host disease, leishmaniosis, allograft rejection, staphylococcus aureus infection and type I diabetes mellitus were up-regulated. Our KEGG pathway enrichment analysis of the DEGs showed that genes involved in oxidative phosphorylation (*pyro phosphatase* and *ATPase H+ transporting V0 subunit a4*) and Jak-STAT signaling (*colony stimulating factor 3*) were up-regulated after knockout of the *MSTN* gene (Fig 5).

### Verification of DEGs by qRT-PCR

**Fig 6:**
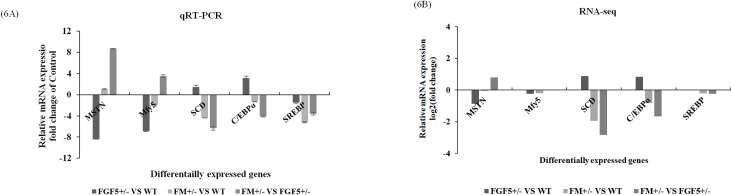
Relative expression levels of MSTN, SCD, C/EBPα, SREBP and Mfy5 determined by qRT-PCR and RNA-Seq. A: qRT-PCR; and B: RNA-Seq.

To further confirm and validate the transcriptome analysis results, the RNA samples isolated for RNA-Seq were used in the qRT-PCR analysis. The DEGs were selected based on the expression profiles and the following criteria: DEGs with a fold-change (log2) ≥ 1 or fold-change (log2) ≤ -1 and a *P*-value < 0.05. A subset of 5 genes selected from the list of DEGs was selected for qRT-PCR analysis. These genes were associated with muscle structure [myogenic actor 5 (*Mfy5*)], muscle fat content [stearoyl coenzyme A dehydrogenase (*SCD*), CCAAT-enhancer-binding protein *α* (*C/EBPα*) and sterol-regulatory element binding proteins (*SREBP*)] and muscle growth/differentiation (*MSTN*). Overall, the qPCR results showed good correspondence with the transcriptome analysis results, indicating that the RNA-Seq data were reliable and accurate (Fig 6). The discrepancies with respect to the ratio should be attributed to the different algorithms and sensitivity of the two techniques [73, 74].

## Discussion

Myostatin is a powerful negative regulator of skeletal muscle growth and development. Although myostatin inhibition in skeletal muscles is valuable for agricultural applications, the molecules downstream of myostatin in skeletal muscle have not been fully identified [75]. Transcriptome analysis is an important method of exploring functional genes and is both the foundation and starting point for studying gene functions and structures [76]. Previous studies have not explored the transcriptome profile of the *MSTN* knockout goat. To our knowledge, this is the first study to investigate the effects of *MSTN* knockout on muscle hypertrophy and functionality as well as whole-body lipid metabolism in a goat model.

*FGF5* resides on chromosome 4q21.21, directly within the region of homozygosis [77]. FGF5, which is a member of the FGF family and has 23 related genes, regulates hair length in humans and a variety of other animals. The *FGF5* family is involved in the control of numerous physiological processes, including embryonic development, neuronal survival, wound repair and angiogenesis, but is also involved in a number of pathological responses [78–80]. In postnatal muscles, satellite cells are the myogenic precursors and are situated beneath the myofiber basement membrane. FGF family proteins can maintain the proliferative state of satellite cells in rat myofiber cultures; specifically, *FGF1*, *FGF4*, and *FGF6* enhance satellite cell proliferation, whereas FGF5 and FGF7 are ineffective [81]. Mice that are homozygous null for *FGF5* display no obvious defects in limb or axial muscle development but are characterized by substantial hair overgrowth. This finding, which is an important demonstration that *FGF5* is not required for normal limb development [82], provides evidence that *FGF5* is not involved in muscle development. Recent studies have suggested that the *FGF5* gene is associated with hair length and in controls the cessation of the anagen stage. *FGF5* signaling pathway is binding of canonical FGFs to FGFR with heparin sulfate as a cofactor induces the formation of ternary FGF—FGFR-HS complex, which activates the FGFR intracellular tyrosine kinase domain by phosphorylation of specific tyrosine residues. The activated receptor is coupled to intracellular RAS-MAPK signaling pathway [83, 84]. However, *MSTN* is a negative regulator of skeletal muscle development and growth, and it is expressed mainly in muscle. The myostatin-Smad pathway alters the activity of protein kinase AKT, thereby inhibiting mTOR pathway and protein synthesis [85]. Therefore, hitherto, no works have yet addressed the possible interaction between *MSTN* and *FGF5*. Taken together, our results support that *FGF5* has not affect the function of *MSTN*. We assessed the function and molecular mechanism of introducing myostatin dysfunction in *FGF5* knockout goats in order to compare the differences between *FGF5/MSTN* dual knockout and *FGF5* single knockout goats.

Myostatin dysfunction results in a dramatic increase of animal muscle mass due to increases in both the numbers and cross-sectional areas of myofibrils [7, 86]. In the present study, we were the first to use transcriptome analysis to identify genes expression changes caused by the knockout of *MSTN* in goats. In total, 68.93 Gb of clean data were obtained. Several DEGs involved in muscle development, fatty acid metabolism, glucose metabolism and oxidative phosphorylation were identified following *MSTN* and *FGF5* gene knockout; these results may benefit studies of the function and molecular mechanism of myostatin in goats. Changes in the downstream molecules of *MSTN*, including the increased expression levels of *MYH15* (myosin heavy chain 15), *MAPKAPK3* (mitogen-activated protein kinase-active protein kinase) and *MYOZ3* (myozenin 3) [87–89], indicated that functional *MSTN* activity or expression was enhanced in FM +/- goats compared to that in the FGF5 +/- and WT goats. Myostatin expression is restricted to developing skeletal muscles, but myostatin protein is still expressed and secreted by skeletal muscles in adulthood [90, 91]. Though the effects of *MSTN* knockout varies by age, no differences were found in body size and weight at birth among homozygous KO lambs and their WT counterparts. However, KO lambs were 20–30% heavier than WT lambs 60 days later despite having a similar body size [92, 93]. The causes of these results are not entirely understood, but it is clear that these results are dependent on the genetic background.

This study showed that genes related to the immune system were up-regulated in the FGF5^+/-^ group compared with the WT and FM^+/-^ groups. Overexpression of *KRT27* has been reported in the inner root sheath [52], and *KRT27* has been shown to regulate innate immune functions [94]. Furthermore, cell adhesion pathway enrichment analysis of the DEGs revealed changes in the intercellular adhesion molecule 3 (*ICAM-3*), human leukocyte antigen (HLA) class II histocompatibility antigen, BOLA class I histocompatibility antigen and HLA class II histocompatibility antigen genes. *ICAM-3*, a member of the *ICAM* immunoglobulin family of adhesion molecules, binds to leukocyte function antigen and mediates the initial localization of neutrophils to sites of tissue injury and inflammation in autoimmune diseases [95]. CAMs of the immunoglobulin superfamily nucleate and maintain groups of cells at key sites during early development and in the adult stage. In addition to their adhesive properties, the binding of CAMs can affect intracellular signaling and developmental events, including cell migration, proliferation, and differentiation [96].

Our KEGG pathway enrichment analysis of the DEGs showed that the skeletal muscle growth, fatty acid metabolism, glucose metabolism and oxidative phosphorylation pathways were enriched in DEGs after *MSTN* knockout. The DEG *HES1*, which is involved in Notch signaling, may act as a regulator of myogenesis by inhibiting the function of *MyoD1* and *ASH1*. It is well known that AMPK acts as a key energy sensor that balances anabolism and catabolism by monitoring either cellular ATP levels or glycogen content.

The MAPK signaling pathway was significantly enriched in DEGs (MAPKAPK3, RAC2, CDC25B and PLA2G4E) after knockout of the *MSTN* and *FGF5* genes. Myostatin activates Erk1/2 MAPK in both proliferating and differentiating C2C12 cells [97]; thus, *MSTN* may affect muscle structures in goats. The MAPK/ERK pathway is reported to be associated with cell proliferation, differentiation, migration, senescence and apoptosis [98, 99]. A wealth of data has revealed a cross-talk between myostatin and the intracellular AKT/mTOR signaling pathway, strongly supporting the notion that myostatin affects the muscle protein balance by regulating protein synthesis and degradation [100]. Adult muscle growth is primarily due to the increase of protein content through activation of the AKT/mTOR pathway, which regulates protein synthesis [101]. Inhibition of myostatin, a negative growth modulator in muscles, functionally enhances muscle mass and improves glucose and fat metabolism in myostatin propeptide transgenic mice [102]. Published studies have clearly shown that adipogenesis is decreased in *MSTN* KO mice. Moreover, the absence of *MSTN* results in enhanced peripheral tissue fatty acid oxidation and increased thermogenesis, culminating in increased fat utilization and reduced adipose tissue mass [103]. Consistent with previous studies, our study showed that fatty acid synthesis is decreased in heterozygous *MSTN* goats, and our KEGG pathway enrichment analysis of the DEGs showed that the fatty acid metabolism pathway was significantly enriched in DEGs after *MSTN* knockout in goats.

Both adipocytes and myocytes are derived from the same mesodermal precursor cells during development. C3H10T(1/2) cells, a mesenchymal fibroblast-like cell line of embryonic origin, have the capacity to undergo differentiation into multiple cell lineages, such as myoblasts, chondrocytes, and adipocytes, after incubation in different media in vitro [104–106]. Later, differentiation controlled by different transcriptional pathways yields individual tissues, suggesting that the myostatin gene is involved in regulating both adiposity and muscularity [92]. In a previous study, we used CRISPR/Cas9 technology and single cell embryo injection and showed that these two methods differ in terms of gene editing efficiency. Specifically, single cell embryo injection could result in abnormal fetus development and high fetal mortality, whereas CRISPR/Cas9 technology did not affect the health of the animals [107]. More importantly, the present study identified the response of some important genes, including *TNMD*, *SCD* and *FANS*, to *MSTN* disruption. These results will be beneficial for breeding purposes.

## Conclusions

In this study, we assembled, characterized, and evaluated the muscle tissue transcriptome and quantified the gene expression levels in *MSTN* and *FGF5* gene-modified goats. Through the development of a rigorous multistep bioinformatics approach, the sequencing reads obtained from *MSTN* knockout goats were successfully cleaned of host sequences and were found to contain high numbers of important genes. The results presented here and the associated assembly will help improve our understanding of the *MSTN* gene expression profile and molecular mechanisms. These data are valuable resources for future studies of goat genomics and will be beneficial for breeding applications that target multiple genes with strong effects on economically important traits in animals. Finally, the results of this study suggest that myostatin disruption might be a promising strategy for the treatment of type 2 diabetes and related metabolic diseases.

## Supporting information

S1 Checklist NC3Rs ARRIVE guidelines checklist. (DOCX)

S1 Table Genotypes of six live *MSTN*- and *FGF5*-knockout goats. (DOCX)

S2 Table Primers for quantitative PCR analysis. (DOCX)
