# Supplementary material for: RNA-seq reveals transcriptome changes in goats following myostatin gene knockout
Source: PLoS One. 2017 Dec 11;12(12):e0187966. doi: 10.1371/journal.pone.0187966 (PMC5724853; doi:10.1371/journal.pone.0187966)
Supplement: S1 Checklist — (DOCX) [file pone.0187966.s001.docx]

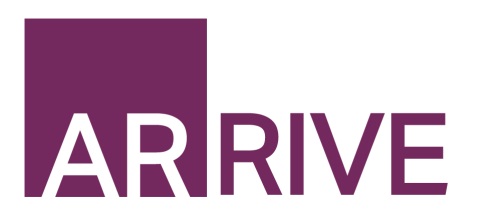


The ARRIVE Guidelines Checklist

Animal Research: Reporting In Vivo Experiments

Lamei Wang, Xiaolong Wang, Bei Cai, Shiwei Zhou, Haijing Zhu^2,3^, Lei Qu ^2,3^, Yulin Chen^1^*

^1^ College of Animal Science and Technology, Northwest A&F University, Yangling 712100, China, ^2^ Shaanxi Provincial Engineering and Technology Research Center of Cashmere Goats, Yulin 719000, China, ^3^ Life Science Research Center, Yulin University, Yulin 719000, China.

| Title 1 **RNA-Seq reveals transcriptome changes in goat****s following myostatin gene knockout** | | ITEM | RECOMMENDATION | Section/ Paragraph |
| --- | --- | --- | --- | --- |
|  | | |  |  |
| Abstract 2 Myostatin (MSTN) is a powerful negative regulator of skeletal muscle mass in mammalian species that is primarily expressed in skeletal muscles, and mutations of its encoding gene can result in the double-muscling trait. In this study, the CRISPR/Cas9 technique was used to edit *MSTN* in Shaanbei Cashmere goats and generate knockout animals. RNA sequencing was used to determine and compare the transcriptome profiles of the muscles from three wild-type (WT) goats, three fibroblast growth factor 5 (*FGF5*) knockout goats (FGF5^+/-^ group) and three goats with disrupted expression of both the *FGF5* and *MSTN* genes (FM^+/-^ group). The sequence reads were obtained using the Illumina HiSeq 2000 system and mapped to the *Capra hircus* reference genome using TopHat (v2.0.9). In total, 68.93, 62.04 and 66.26 million clean sequencing reads were obtained from the WT, FM^+/-^ and FGF5^+/-^ groups, respectively. There were 201 differentially expressed genes (DEGs) between the WT and FGF5^+/-^ groups, with 86 down- and 115 up-regulated genes in the FGF5^+/-^ group. Between the WT and FM^+/-^ groups, 121 DEGs were identified, including 81 down- and 40 up-regulated genes in the FM^+/-^ group. A total of 198 DEGs were detected between the FGF5^+/-^ group and FM^+/-^ group, with 128 down- and 70 up-regulated genes in the FM^+/-^ group. At the transcriptome level, we found substantial changes in genes involved in fatty acid metabolism and the biosynthesis of unsaturated fatty acids, such as stearoyl-CoA dehydrogenase, 3-hydroxyacyl-CoA dehydratase 2, ELOVL fatty acid elongase 6 and fatty acid synthase, suggesting that the expression levels of these genes may be directly regulated by *MSTN* and that these genes are likely downstream targets of *MSTN* with potential roles in lipid metabolism in goats. Moreover, five randomly selected DEGs were further validated with qRT-PCR, and the results were consistent with the transcriptome analysis. The present study provides insight into the unique transcriptome profile of the *MSTN* knockout goat, which is a valuable resource for studying goat genomics. | | |  |  |
| INTRODUCTION | | |  |  |
| \| Background \| 3 \| 1. Myostatin (MSTN) is a secreted growth factor and a member of the TGF-β superfamily. It functions as a critical autocrine/paracrine inhibitor, and it negatively regulates skeletal muscle growth and development through the regulation of anabolic and catabolic pathways in skeletal muscles. Myostatin is expressed almost exclusively in skeletal muscle. Mutations in the coding region of MSTN results in a double-muscling phenotype in many species, including cattle, mice and humans. Natural myostatin gene mutations occur in cattle breeds such as Belgian Blue, exhibiting an obviously increased muscle mass. High-throughput mRNA sequencing (RNA-seq) offers the ability to discover new genes and transcripts and measure transcripts and measure transcript expression in a single experiment 2. The Shaanbei Cashmere goat is a local breed in China that produces both fiber and meat products, The meat is renowned for its lower fat content compared to beef and lamb. All animals were Cas9-mediated gene-modified goats. \| \| --- \| --- \| --- \| | | |  |  |
| Objective 4 The goals of this study were to identify important candidate genes related to muscle, glucose and lipid metabolism and further determine the transcriptome changes using RNA-Seq. | | |  |  |
| METHODS | | |  |  |
| Ethnic statement 5 All animal experiments and procedures were carried out in strict accordance with the recommendations in the Guide for the Care and Ues of Laboratory Animals of the National Institutes of Health. The protocol was approved by the Committee on the Ethics of Animal Experiments of the Northwest A&F University (Approval ID: 2014ZX08008-002). | | |  |  |
| Study design 6 Nine Shaanbei Cashmere goats (one year old), three wild-type (WT) (animals determined to possess an unedited genome) goats, three goats with only the *fibroblast growth factor 5* (*FGF5*) gene knocked out (FGF5^+/-^ group) and three goats with disruptions in both the *FGF5* and *MSTN* genes (FM^+/-^ group). | | |  |  |
| \| Experiment  procedures \| 7 \| 1. Fresh longissimus dorsi muscle samples were harvested immediately from goats after surgery, 3 mL procaine (1%) was injected into skin/muscle tissues for local anesthesia. 2. In the morning. 3. In the farm. 4. All surgeries were strict accordance with the procedures of clinical trial. 5. Using RNA-Seq determine the transcriptome changes. \| \| --- \| --- \| --- \| | | |  |  |
| \| Experiment  animals \| 8 \| 1. Shaanbei Cashmere goat (one year old). 2. The transgenic goats were generated using the CRISPR/Cas9 system. All transgenic goats were heterozygote and healthy. \| \| --- \| --- \| --- \| | | |  |  |

| \| Housing and  husbandry \| 9 \| 1. All animals were raised in the same way in natural lighting with free access to water and food at the Shaanbei Cashmere goat Farm of Yulin University. 2. Research was conducted a protocol in compliance with the Animal Welfare Act. \| \| --- \| --- \| --- \| |  |  |  |
| --- | --- | --- | --- | --- | --- | --- |
| \| Sample size \| 10 \| 1. nine Shaanbei Cashmere goats (one year old), three wild-type (WT) (animals determined to possess an unedited genome) goats, three goats with only the *fibroblast growth factor 5* (*FGF5*) gene knocked out (FGF5^+/-^ group) and three goats with disruptions in both the *FGF5* and *MSTN* genes (FM^+/-^ group). 2. Each group include the number of three goats in compliance with the Principle of Statistics. \| \| --- \| --- \| --- \| |  |  |  |
| \| Allocating  Animals to  Experimental  groups \| 11 \| 1. Each group goats was selected in accordance with the different of knockout gene. 2. Each group goats were randomly treated and assessed. \| \| --- \| --- \| --- \| |  |  |  |
| \| Experimental  outcomes \| 12 \| In total, 68.93, 62.04 and 66.26 million clean sequencing reads were obtained from the WT, FM^+/-^ and FGF5^+/-^ groups, respectively. At the transcriptome level, we found substantial changes in genes involved in fatty acid metabolism and the biosynthesis of unsaturated fatty acids. \| \| --- \| --- \| --- \| |  |  |  |
| \| Statistical  methods \| 13 \| All data are presented as the mean ± standard deviation (SD), and comparisons were performed by analysis of variance (ANOVA) (SAS). A probability of less than 0.05 was considered statistically significant. \| \| --- \| --- \| --- \| |  |  |  |
| RESULTS |  |  |  |
| \| Baseline  data \| 14 \| \| **Group** \| **Gender** \| **Targeting information^a^** \| \| \| **Weight**  **(Kg)** \| **drug** \| **test** \| \| --- \| --- \| --- \| --- \| --- \| --- \| --- \| --- \| \| ***FGF5*** \| ***MSTN*_sg1** \| ***MSTN*_sg2** \| \| FM ^+/-^ \| M \|  \|  \|  \| 38.1 \| NO \| Yes \| \| M \|  \|  \|  \| 35.9 \| NO \| Yes \| \| M \|  \|  \|  \| 36.8 \| NO \| Yes \| \| FGF5^+/-^ \| M \|  \|  \|  \| 29.5 \| NO \| Yes \| \| F \|  \|  \|  \| 24.4 \| NO \| Yes \| \| F \|  \|  \|  \| 22.5 \| NO \| Yes \| \| WT \| M \|  \|  \|  \| 24.7 \| NO \| Yes \| \| M \|  \|  \|  \| 25.1 \| NO \| Yes \| \| F \|  \|  \|  \| 22.4 \| NO \| Yes \| \| \| --- \| --- \| --- \| --- \| --- \| --- \| --- \| --- \| --- \| --- \| --- \| --- \| --- \| --- \| --- \| --- \| --- \| --- \| --- \| --- \| --- \| --- \| --- \| --- \| --- \| --- \| --- \| --- \| --- \| --- \| --- \| --- \| --- \| --- \| --- \| --- \| --- \| --- \| --- \| --- \| --- \| --- \| --- \| --- \| --- \| --- \| --- \| --- \| --- \| --- \| --- \| --- \| --- \| --- \| --- \| --- \| --- \| --- \| --- \| --- \| --- \| --- \| --- \| --- \| --- \| --- \| --- \| --- \| --- \| --- \| --- \| --- \| --- \| --- \| --- \| --- \| --- \| --- \| --- \| --- \|   ^a^The shadows indicate the occurrence of disruption at given locus. |  |  |  |
| \| Numbers  analysis \| 15 \| 1. In each group include three goats in each analysis. 2. No modifications to the experimental protocols. \| \| --- \| --- \| --- \| |  |  |  |
| \| Outcomes and  estimation \| 16 \| \| Name \| *FM^+/- VS WT^* (SD) \| *FGF5^+/-^* (SD) \| WT (SD) \| \| --- \| --- \| --- \| --- \| \| MSTN \| 0.072315 \| 0.009286 \| 0.032471 \| \| Mfy5 \| 0.1947 \| 0.030062 \| 0.230899 \| \| SCD \| 0.011685 \| 0.382664 \| 0.341724 \| \| C/EBPα \| 0.044806 \| 0.380023 \| 0.097572 \| \| SREBP \| 0.07875 \| 0.118581 \| 0.215938 \| \| \| --- \| --- \| --- \| --- \| --- \| --- \| --- \| --- \| --- \| --- \| --- \| --- \| --- \| --- \| --- \| --- \| --- \| --- \| --- \| --- \| --- \| --- \| --- \| --- \| --- \| --- \| --- \|   SD: standard deviation. |  |  |  |
| \| Adverse events \| 17 \| No important adverse events. \| \| --- \| --- \| --- \| |  |  |  |
| DISCUSSION |  |  |  |
| \| Interpretation/  Scientific  implication \| 18 \| 1. Myostatin dysfunction results in a dramatic increase of animal muscle mass due to increases in both myofibril numbers and myofibrillar cross-sectional area. In the present study, we were the first to identify genes expression changes caused by the knockout of *MSTN* in goats by transcriptome analysis. 2. Our studies are not to generate more enough homozygote *MSTN* goat to analyze and clarify the involved mechanisms of *MSTN*-mediated muscle development. 3. No any implications of our experimental methods or finding for the 3Rs of the ues of animals in research. \| \| --- \| --- \| --- \| |  |  |  |
| \| Generalisability/  translation \| 19 \| It is a valuable resource for future studies of goat genomics and will benefit breeding applications by targeting multiple genes with strong effects on economically important traits animals. In addition to its antisarcopenic effects, these results would suggest that disrupted myostatin may also be a promising strategy for the treatment of type 2 diabetes and related metabolic disease. \| \| --- \| --- \| --- \| |  |  |  |
| \| Funding \| 20 \| This work is supported by National Natural Science Foundation of China (31372279, 31171377, 31572369) to YC, and China Agriculture Research System (CARS-40-13) to YC, as well as the Major Projects for New Varieties of Genetically Modified Organisms of China (2014ZX08008-002) to YC, National Natural Science Foundation of China (31402038) and the key Research Program of Shaanxi Province (2017NY-072) to XW, and the Special Fund for Agro-scientific Research in the Public Interest (201303059) to YC. The funders had no role in study design, data collection and analysis, decision to publish, or preparation of the manuscript. \| \| --- \| --- \| --- \| | |  | |
