# Supplementary material for: RNA-seq reveals transcriptome changes in goats following myostatin gene knockout
Source: PLoS One. 2017 Dec 11;12(12):e0187966. doi: 10.1371/journal.pone.0187966 (PMC5724853; doi:10.1371/journal.pone.0187966)
Supplement: S1 Table — (DOCX) [file pone.0187966.s002.docx]

**S1 Table. Genotypes of six alive *MSTN*- and *FGF5*-disrupted goats.**

| **Group** | **No.** | **Animal**  **ID (#)** | **Gender** | **Targeting information^a^** | | | **Mutation at**  **MSTN_sg1 locus** | **Mutations at MSTN_sg2 locus** |
| --- | --- | --- | --- | --- | --- | --- | --- | --- |
|  |  |  |  | ***FGF5*** | ***MSTN*_sg1** | ***MSTN*_sg2** | **Genotype^b^** | **Genotype** |
| FM ^+/-^ | 1 | #9 | M |  |  |  | -12bp |  |
|  | 2 | #40 | M |  |  |  |  | -1bp |
|  | 3 | #70 | M |  |  |  |  | -4bp |
|  |  | |  |  | | | Mutation in *FGF5* locus (Genotype) | |
| FGF5^+/-^ | 4 | #19 | M |  |  |  | +1m2bp, -21bp, -41m6bp | |
|  | 5 | #21 | F |  |  |  | -218+28bp, -26bp, -57bp, +10bp | |
|  | 6 | #53 | F |  |  |  | -43bp | |

^a^The shadows indicate the occurrence of disruption at given locus.

^b^‘-n’ indicates n bp deletion, ‘+n’ indicates n bp insertion, ‘m’ represents the number of point mutations.
