# Supplementary material for: RNA-seq reveals transcriptome changes in goats following myostatin gene knockout
Source: PLoS One. 2017 Dec 11;12(12):e0187966. doi: 10.1371/journal.pone.0187966 (PMC5724853; doi:10.1371/journal.pone.0187966)
Supplement: S2 Table — (DOCX) [file pone.0187966.s003.docx]

**S2 Table. Primers for quantitative PCR analysis.**

| Genes | Forward primers ( 5＇ 3＇) | Reverse primers ( 5＇ 3＇) |
| --- | --- | --- |
| MSTN [47] | GTGTTGCAAAACTGGCTCAA | TCATCACAATCAAGCCCAAA |
| Myf5 [48] | GCAAGAGGAAGTCCACCAC | CAGCCTCTGGTTAGGGTTG |
| SCD [49] | GTGATGTTCCAGAGGAGGTACTACAA | AACGTTTCATCCCACAGATACCA |
| SREBP [49] | CCGAGGCCAAGTTGAATAAATCT | ACACCAGGTCCTTCAGCGATTTG |
| C/EBPα [49] | GCAAAGCCAAGAAGTCCG | GGCTCAGTTGTTCCACCCGCTT |
| GAPDH [47] | ATGTTTGTGATGGGCGTGA | AAGCAGGGATGAAGTTCTGG |
